# Impact of Dietary Protein on Osteoporosis Development

**DOI:** 10.3390/nu15214581

**Published:** 2023-10-28

**Authors:** Gabriela Kędzia, Martyna Woźniak, Włodzimierz Samborski, Bogna Grygiel-Górniak

**Affiliations:** Department of Rheumatology, Rehabilitation and Internal Diseases, Poznan University of Medical Sciences, 61-701 Poznan, Poland

**Keywords:** osteoporosis, dietary protein, protein intake, dairy and soy products, bone health

## Abstract

Osteoporosis is a frequent yet unsolved health problem among older people. The influence of dietary protein still raises many questions regarding its quality and quantity in the context of bone health. The aim of this manuscript is to review the latest evidence on plant and animal protein influences on bone health in various groups of patients. The review is based on original studies, meta-analyses, randomized controlled trials, and prospective cohort studies published in PubMed and Cochrane databases during the last five years. Combining plant and animal protein with physical activity has the best effect on bones (muscle strengthening and reducing the risk of falls), while high protein intake can have adverse effects during bed rest. Despite the content of isoflavones, plant protein is not more beneficial than animal protein (dairy products) and can increase bone resorption markers. Hypoestrogenism due to menopause or eating disorders leads to low bone density and an increased risk of osteoporosis. A well-balanced diet with sufficient energy supply and protein intake (both of plant and animal origins) and adequate physical activity are crucial to ensure bone health. Dietary interventions should consider the quantity and quality of protein in patients with other comorbidities, particularly in an aging society.

## 1. Introduction

Osteoporosis is one of the most prevalent diseases, severely burdening the healthcare system. Many risk factors are associated with this disease. One of them is advanced age [1]. The increasing number of older adults in the general population and the growing availability of diagnostic procedures impact osteoporosis prevalence. The risk of osteoporosis among American adults above 50 increased from 9.4% in 2007–2008 to 12.6% in 2017–2018, and the disease predominated the female gender [2]. In 29 European countries, approximately 32 million people in 2019 were diagnosed with osteoporosis (6.5 million men and 25.5 million women). Women are obviously at a higher risk of osteoporosis, due to postmenopausal estrogen decline [3].

In the course of osteoporosis, the remodeling of bone tissue is disrupted in favor of bone resorption. Decreasing osteoblasts’ activity leads to a structural change in bone structure and a diminished density [4]. Unfortunately, severe or untreated osteoporosis eventually leads to osteoporotic fracture triggered by the energy that would not cause bone trauma in a healthy subject [1]. Globally, in 2010, there were 158 million patients over 50 years old with a high risk of osteoporotic fracture. A recent calculation shows that in 2040, this number can double to over 300 million [5].

Since osteoporosis is a challenge to the modern healthcare system, many scientific approaches have been made to estimate the possible risk factors and protective behaviors on bone condition. In clinical practice, diagnostic methods of specific osteoporosis markers are broadly discussed. A lot of data highlighted the meaning of healthy behavior, including adequate calcium and vitamin D consumption [6,7]. However, without proper protein intake, bone mass decreases much quicker than in the diet without sufficient calcium and vitamin D supplementation. Protein is a fundamental macronutrient, which should be analyzed because it accounts for 50% of the bone’s volume and a third of its mass. It influences collagen and hormone syntheses, participating in the building of bone mass [8]. Moreover, protein reveals anabolic activity by increasing insulin-like growth factor 1 (IGF-1) and stimulating bone growth [9,10,11].

On the other hand, some studies report the potentially destructive influence of protein on the bone, which can increase calcium excretion; however, these adverse effects are only observed in patients with renal insufficiency [12,13]. Thus, the quantity and quality of protein (plant or animal) still raise many questions. This manuscript broadly discusses the role of protein in the development and treatment of osteoporosis. The available data on the patient’s age, physical activity (PA), and co-existing diseases (cardiovascular, kidney, and eating disorders-ED) are also analyzed.

## 2. Materials and Methods

**Figure 1:**
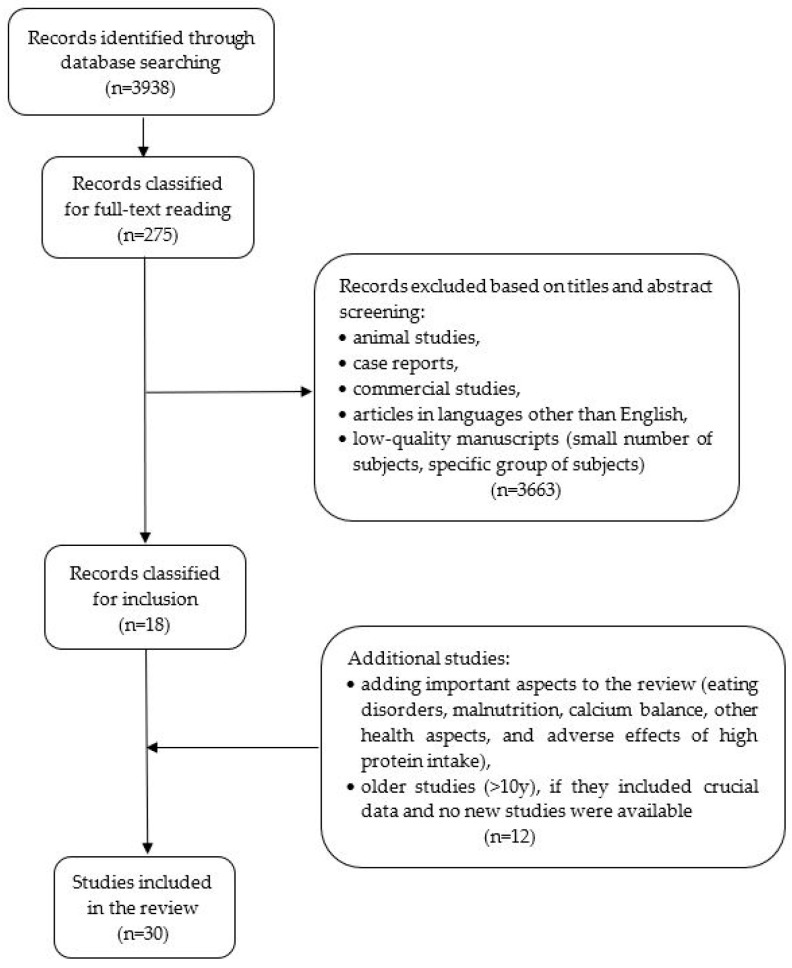
Flowchart of the selection of the studies included in the present review.

The review was based on an analysis of original studies, meta-analyses, randomized controlled trials (RCT), and prospective cohort studies published in the PubMed database (Figure 1). The keywords included the words: osteoporosis, osteoporosis and dietary protein, osteoporosis and plant/animal protein, osteoporosis and collagen, dairy products, protein intake, intake, bone health, peptide metabolism, and bone mineral density. The exclusion criteria included low-quality manuscripts (small number of subjects and specific group of issues making it impossible to conclude the general population), case reports, and articles in languages other than English. It was decided to include papers from the last 10 years as the most recent and detailed. Older studies were included if they presented crucial data, and no new studies on that subject have been published in recent years. The manuscripts that had the highest number of patients, adequate osteoporosis diagnosis, and the most precise estimated dietary components (intake of specific nutrients) were comprised in this analysis.

## 3. Results

### 3.1. Amount of Protein in the Diet and the Risk of Osteoporosis

Recently, many studies have analyzed the effect of protein quantity on bone mass. The recommended protein amount in the diet should be estimated based on gender, age, body mass, and possible protein sources in the traditional diet. According to the Institute of Medicine, the recommended dietary allowance (RDA) of protein is estimated at 0.8 g/kg of body weight [14]. However, scientific curiosity raises the question of whether a high-protein diet (above the RDA) has a harmful impact on bone health. For example, Finnish studies suggest that excess protein intake in the national diet can have a negative effect on bone mineral density (BMD) and bone mineral content (BMC). This prospective cohort study revealed that increased protein intake (>1.2 g/kg body mass/day) by postmenopausal, non-active women for three years is negatively correlated with BMD and BMC [15]. Similarly, a high-protein diet (1.45 g/kg/day) causes an increase in CTXI (C-terminal telopeptide of type I collagen, a marker that intensifies the decrease in bone mass) in bed-rest women. Such influence is observed even if appropriate calcium and potassium quantities are delivered with the isocaloric diet [16].

On the contrary, other data show the protective effect of protein on bone remodeling, even consumed in excess, in patients without specific dietary intervention but with increased PA [15,17]. A meta-analysis of Wallace and Frankenfeld revealed a possible reduction in hip fractures when protein intake was over RDA (0.8 g/kg/day). In prospective cohort studies, the dietary intake was assessed with a validated food frequency questionnaire (FFQ), 24 h dietary recall, or food records. This meta-analysis showed that a higher dietary protein intake prevents BMD loss [18]. Similarly, the prospective analysis conducted by the Women’s Health Initiative (WHI), including 140,000 women (50–79 year), showed an increased total BMD in the group that consumed more protein (20% total energy). The increased protein consumption promoted the longer maintenance of a higher BMD [19]. A comparable effect was observed in pairs of monozygotic twins. One of the twins’ higher daily protein intake resulted in a significantly higher BMD value of the spine [20]. Conversely, a meta-analysis of the American population revealed no statistically significant beneficial effect of a higher protein intake on BMD. There was only a slight improvement in lumbar spine BMD, and no impacts on total hip BMD, femoral neck BMD, or bone markers were described [21]. All the mentioned analyses raised the question of whether increased BMD can influence the final risk of fractures. The WHI study showed that the increase in BMD was not significantly related to hip and spine fractures, but it had a protective effect on the number of forearm fractures [19].

Other than quantity, protein quality also determines BMD value. For example, specific amino acids such as alanine, arginine, glutamic acid, leucine, lysine, and proline positively affect BMD [20]. Since available data are ambiguous, nutritional interventions should be careful. The quantities of plant and animal proteins must be precisely calculated while paying attention to gender, age, body mass, ethnicity of patients, and official nutrition guidelines and recommendations (specific to each national diet). 

### 3.2. Dietary Protein and Calcium Balance

For many years, the scientific debate has tried to resolve a controversial issue: whether a high-protein diet increases bone calcium resorption. It has been suggested that even if increased protein consumption causes excessive calciuria, it probably does not decrease the overall calcium level. Furthermore, such a diet does not increase the resorption of this mineral from the bones [22,23]. This fact results from co-existing increased intestinal calcium absorption in case of excessive protein intake [23]. Moreover, after higher meat intake, hypercalciuria is transient, with an adaptation time of about three weeks [24]. Thus, excessive calcium excretion caused by high protein intake does not seem to cause bone mineral loss.

### 3.3. Effect of Protein Supplementation on Bone Markers

Protein supplementation affects bone mineral density measured by densitometry and metabolic parameters of bone quality, such as bone formation markers: bone-specific alkaline phosphatase (BSAP), osteocalcin (OC), and N-terminal propeptide of type I procollagen (PINP); and bone resorption markers: CTX, pyridinoline cross-linked collagen polypeptide, and N-terminal telopeptide of type I collagen (NTX). According to the International Osteoporosis Foundation, PINP and CTX are the most useful and reliable markers for monitoring osteoporosis [25]. These markers are used in clinical and nutritional studies and are analyzed in various diets [16,26,27]. For example, a decreased carbohydrate intake with associated elevated protein consumption positively affects bone parameters. Such a diet causes a reduction in NTX in postmenopausal women with osteopenia. Conversely, for low-carbohydrate consumption, the low-protein diet has an unbeneficial influence on bone markers in females and causes decreased OC and increased NTX levels [26].

Another parameter often used in the estimation of osteoporosis risk is IGF-1. This anabolic hormone plays an essential role in bone formation [28]. Dietary protein intake has been shown to increase the secretion of IGF-1, particularly in soy products. Moreover, the elevation of IGF-1 concentration is significantly higher when compared to casein intake [10]. In addition, according to Bonjour et al., soft, white cheese fortified with calcium and vitamin D consumed for 6 weeks by postmenopausal women (50–65 year) with a low risk of fracture resulted in a reduced level of the resorption marker TRAP5b (tartrate-resistant acid phosphatase). This marker is correlated with increased IGF-1 and increased osteoblasts activity [29].

The influence of high protein varies in the case of the quantity of supplied energy. A low-energy diet, which provides between 800 and 1200 kcal/day, is used in treating severe obesity [30]. Such a diet alters bone markers to increase resorption by changing leptin and IGF-1 levels. A low serum leptin concentration is associated with an increased risk of fractures [31]. This process is observed during long training sessions of endurance athletes (e.g., cyclists), who are particularly vulnerable to low energy availability, even if not on a restricted diet. As a result, impaired bone metabolism by increasing bone resorption and an increased risk of bone fractures are observed [30,31]. However, in the study of Murphy et al., the high protein supply by athletes during training did not impact bone turnover markers (IGF-1, leptin, CTX-I, P1NP, sclerostin, and IGFBP-3 (insulin-like growth factor-binding protein-3) [31].

### 3.4. Dairy Products in the Prevention and Treatment of Osteoporosis

Dairy products contain high-quality protein and elements substantial for bone health, such as calcium, vitamin D, phosphorus, and potassium. Three recent meta-analyses emphasized the impact of milk and dairy product consumption on osteoporosis and fracture risk (Table 1). Two of them supported the positive effect of protein-rich products (milk and milk beverages) on BMD [32,33]. The dietary intakes in this meta-analysis were measured by FFQ, 3-day or 4-day 24 h dietary recall, a posteriori methods, and phone calls [32]. Dietary patterns named by the authors as “milk/dairy” resulted in a decreased risk of low BMD in every age group [32]. The beneficial influence of dairy products on BMD is more prominent in women from countries with low calcium consumption, e.g., China, India, Argentina, the Republic of South Africa, and others [33,34]. Additionally, fortified dairy products may trigger a more favorable effect on bone metabolism than only calcium supplements because they contain other nutritional components that augment calcium absorption in the intestines, e.g., aromatic amino acids [23,35,36,37].

On the contrary, the results of the meta-analysis of Malmir et al. did not show the benefits of a diet rich in dairy foods (assessed by FFQ in most of the analyzed cohort studies presented in this review). Such a diet did not reduce the risk of osteoporosis and hip fracture [38]. The reason for the discrepancy in the presented data can be connected to the broad range of participants (e.g., youth, adults [32,38], or healthy postmenopausal women [33]).

### 3.5. Plant Protein in the Risk of Osteoporosis

A question that often arises among physicians and nutritionists concerns the importance of plant and animal proteins in developing osteoporosis. The study of George et al. tried to answer this question by analyzing the differences between animal and plant protein intakes in a group of 135 men and women. The participants were supplemented with 40 g of soy protein intake vs. 40 g of casein daily for three months. Plant protein contained soy products, which delivered about 96 mg of isoflavones daily. The study showed that the parameters of bone metabolism, such as the BALP (bone alkaline phosphatase) and TRAP, were unaffected by the diet; however, a beneficial increase in IGF-1 in the soy-supplemented subjects was observed [11].

More detailed data analyzed the effects of protein administration in varying proportions of the plant-to-animal ratio: 30:70, 50:50, or 70:30. After 12 weeks, bone markers such as CTX, parathyroid hormone (PTH), and PINP were significantly higher in the group consuming a higher quantity of plant protein. These markers reflect the increased activity of osteoclasts and are related to increased bone turnover. Additionally, the high-plant diet did not contain the recommended amount of calcium and vitamin D [27]. Thus, a plant protein-rich diet increases bone turnover and does not contain other essential nutrients ensuring skeleton health (Table 2).

**Figure 2:**
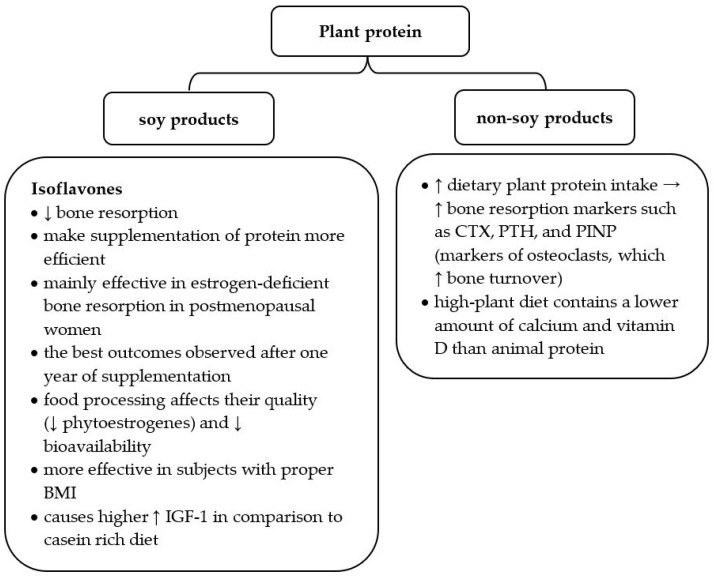
Characteristics of plant protein in the context of osteoporosis risk. BMI—body mass index; IGF—insulin-like growth factor 1; CTX—C-terminal telopeptide of type I collagen, PTH—parathyroid hormone, PINP—N-terminal propeptide of type I procollagen; ↑ increase; ↓ decrease [10,27,39,40,41].

Since soy products are a good source of plant protein and contain beneficial isoflavones, recent data profoundly analyzed the influence of soy food on osteoporosis development (Figure 2). Many studies underline that isoflavones may reduce bone resorption and make protein availability more efficient [39,42,43]. Their structure is similar to estrogens; thus, they positively influence estrogen-deficient bone resorption in postmenopausal women [42]. One meta-analysis proved that soy isoflavones provide the best outcomes after one year of supplementation and are more effective in subjects with a proper body mass index (BMI) [43]. When recommending soy products to patients with osteopenia or osteoporosis, it is worth underlining that food processing affects their quality. Food production changes the percentage of particular nutrients (e.g., phytoestrogens) and influences their bioavailability [39].

The beneficial effect of isoflavones was confirmed by the study of Li et al., revealing a significantly increased lumbar spine BMD after two years of 100 g/day of dried bean curd supplementation in postmenopausal women (64.4 mg/day of isoflavone, RCT study). However, no change was found in laboratory parameters, such as BALP, bone Gla protein (a bone formation marker and one of the bone matrix proteins), and BMD of the right proximal femur [44].

Thus, despite the content of isoflavones, supplementation with plant protein is not more effective than the protein of animal origin. This fact was confirmed by the large meta-analysis of the National Osteoporosis Foundation, which showed no statistically significant differences between the plant-protein-rich diet and the diet with a predominance of animal protein [9].

### 3.6. Protein Supplementation Combined with Physical Activity

The effect of high protein intake is strictly related to lifestyle (including exercise). Thus, the possible beneficial/adverse effects should be analyzed in the context of the daily activity of each patient. For example, an increased protein intake in patients during bed rest causes a rise in bone resorption markers. These markers are related to osteoclast activity and a greater risk of bone loss [16]. On one side, the diet in non-active osteoporotic patients should be carefully planned. On the other side, scheduling PA in older people (who usually have diminished daily activity) plays a crucial role in preventing and managing osteoporosis [17]. It was proved that adequately selected exercises are safe for people diagnosed with osteoporosis and osteopenia [41]. They increase muscle strength and enhance coordination, thus reducing the risk of falling (the leading cause of osteoporotic fracture) [45].

Intriguing is whether PA combined with the increased supply of protein can provide better results in decreasing the risk of osteoporosis. However, studies that examine the above association are limited. One of the studies analyzed a 36-week exercise program in obese adults (35–65 years). The exercise program included resistance training (2 d/wk) and aerobic exercises (1 d/wk). Patients were divided into four groups, each receiving a different dose of protein. This study showed that the protein supplementation associated with specific training did not influence BMD or BMC in obesity [46].

Conversely, a prospective study of postmenopausal women showed that high protein intake (>1.2 g/kg body mass/day) negatively influenced BMD and BMC. However, increased lumbar spine BMD and femoral neck BMC were observed in the subpopulation of women exercising [15]. Thus, PA can diminish the potential negative impact of protein intake on bone mineralization in inactive patients.

As mentioned above, the lack of PA in patients under bed rest causes a decline in bone mass. Therefore, it seems reasonable to supplement protein to prevent this process. Such a hypothesis was studied by Heer et al. in a group of women on a high-protein diet (1.45 g/kg/day) with appropriate micronutrient intake (calcium and potassium, branched-chain amino acids). In this study, subjects were given a weighted quantity of protein daily, and the leftovers were also measured and deducted from their daily intake. Surprisingly, the authors observed the opposite of the suspected results. After 20 days of such supplementation, an increase in CTX was detected in the analyzed physically inactive group of women [16].

The positive effect of PA was also confirmed in the study of Shenoy et al., who analyzed the influence of soy protein isolate (40 g/day) combined with resistance training (4 times/week for 12 weeks) on BMD in postmenopausal women (45–65 y). Such management improved muscle strength and increased BMD, which was measured by quantitative ultrasound densitometry in the radius and tibia [47].

The results of the above studies suggest that high protein supplementation may have a negative impact on the quality of bones and BMD if not associated with PA. Thus, proper PA associated with protein supplementation seems to have positive results on bone mass. Whether these results are achieved due to training itself (which eliminates the negative impact of protein) or combined diet and exercise interventions (which increase each other’s synergistic effectiveness) is still an unresolved question. Hopefully, future studies will answer it, but regardless of the scientific conclusions, PA and a healthy diet are still the best solutions for maintaining good bone health.

### 3.7. Dietary Protein Interventions and Bone Health in Eating Disorders

There are ongoing discussions about whether obesity has a predominantly positive or negative effect on bone health. On one side, body weight increases the mechanical loading of bones, which positively impacts BMD and BMC. However, excess body fat, which contains hormonally active molecules, causes potentially harmful systemic inflammation. (Increased proinflammatory cytokine levels impair calcium absorption) [48]. Additionally, being overweight and obese is associated with the risk of many chronic diseases (hypertension, coronary heart disease, diabetes, etc.) [49].

It has been hypothesized that increased protein intake during body mass reduction would allow for maintaining bone quality and density. The study by Weaver et al. of elderly obese adults (65–79 y) who underwent six months of a hypocaloric high-protein diet (over 1 g/kg/day) showed no negative impact on bone health. Adults from the control group who did not undergo any dietary intervention were encouraged to maintain their weight with a recommended dose of protein (∼0.8 g/kg/day). The dietary assessment method of the experimental group included self-reports provided by participants, 24 h urinary nitrogen-estimated protein intake at baseline and at 6 months, and regular body mass measurement. After successful body mass reduction, the BMD was similar in both groups [50]. The study of Wright et al. showed that consuming more protein during weight reduction in obesity may slightly alleviate BMD loss (observation based on the comparison between the low-energy diet vs. the low-energy diet with a higher quantity of protein) [51]. However, this hypothesis should be confirmed in long, prospective studies.

It is worth emphasizing that a low body mass increases the risk of weakening bone strength [52,53]. This effect is particularly potentiated in excessive body mass loss, as in anorexia nervosa (AN), one of the eating disorders (ED). Various studies report an augmented risk of fracture, osteopenia, and osteoporosis in anorexia [54,55,56]. The precise mechanism of reduced BMD in AN is not yet known; however, low body mass, nutritional restriction, the duration of the disease, and amenorrhea (due to low estrogen levels) contribute to the low BMD in this disease [54,55]. Compared to healthy subjects, patients with AN have a 150–300% higher fracture risk [54].

There are conflicting data about other EDs and BMD. However, bulimia nervosa (BN) might also result in lower BMD, which can be explained by the previous history of AN in patients suffering from BN and prolonged malnutrition observed in BN, disrupted by compulsive eating episodes. Nevertheless, further research is needed to prove this theory [54]. In conclusion, patients with EDs should be screened for BMD and adequately treated to prevent reduced BMD and osteoporosis development [54,55].

## 4. Discussion

Recommendation of an appropriate diet in patients with osteoporosis is often a challenge. Patients with osteoporosis are usually older and present various comorbidities, such as cardiovascular disease (CVD) and kidney disorders. In these patients, indicating an adequate diet with the proper quantity and protein quality is challenging for physicians and dietitians. It was proved that choosing a good source of protein is essential for CVD development. Song et al., in a prospective cohort study, investigated the connection between the protein sources in the diet and all causes of death (including CVD). The results showed that high animal protein intake increases CVD mortality, particularly if it is derived from red meat, eggs, and high-fat dairy products. In contrast, plant protein intake reduces CVD and all-cause mortality. However, a statistically significant reduction in cardiologic risk was observed only in patients with at least one of the following lifestyle risk factors: smoking, alcohol consumption of over 14 g/day, low PA, overweightness, and obesity. Therefore, plant protein or low-fat animal products (such as poultry, low-fat dairy products, and fish) should be included in the diet of patients with CVD risk factors [40].

Another disease, which is also highly prevalent with increasing age, is kidney dysfunction. The source of protein, rather than the total protein intake, impacts the incidence of chronic kidney disease (CKD) or progression to end-stage renal disease (ESRD). Red meat increases the risk of CKD and ESRD, possibly due to its higher acid load. It is suggested that replacing at least one portion of red meat per day with other protein sources may reduce this risk [12,13]. In addition, the intake of whey protein (WP) above 0.8 g/kg did not have a negative effect on the estimated glomerular filtration rate (eGFR) in the elderly over 60 years old without kidney disorders who had normal renal function before protein supplementation [57].

Another risk factor for reduced BMD and osteoporosis is menopause. Due to hypoestrogenism, women after menopause are at a higher risk of osteoporosis; hence, they should be educated and diagnosed in every case of suspected osteoporosis. It seems that plant protein is a better source of protein, but it does not have a better effect on bones than animal protein. Women should also be encouraged to implement adequate PA into their daily habits [9]. In addition to a proper diet, vitamin D supplementation, PA, and pharmacological treatment should be individually considered (e.g., bisphosphonates, hormonal therapy, etc.) [58].

Excessive exercise can lead to low estrogen levels and menstrual disorders in young female athletes, including delayed menarche and functional hypothalamic amenorrhea (FHA). Hypoestrogenism increases the risk of low BMD, osteoporosis, and fractures, including stress fractures. However, future studies are needed to evaluate the prevalence of stress fractures in female athletes and the risk factors in this group with detailed dietary habits assessments, including analysis of protein intake. Nowadays, more and more women get involved in intense physical activity; therefore, bone density should be carefully monitored in this group of active women from a young age [59]. Stress fractures are a common problem in subjects who participate intensively in sports, such as soldiers and athletes. In such groups, a diet rich in protein and minerals can be beneficial [60]. Prospective case-control studies are needed to confirm which dietary behaviors require immediate modifications to prevent future bone fractures.

Determining the exact effect of protein intake on bone density is challenging for several reasons. First, available studies analyze dietary habits in various populations using national diets, which differ in the amount of consumed nutrients and the presence of specific food additives influencing protein absorption. Multiple methods, including daily intake of nutritional products, preferences, or frequency questionnaires, are used to analyze protein intake. Therefore, comparing the effects of individual diets on bone health is often impossible. Another limitation is protein consumption from different dietary courses undergoing specific culinary processing. Moreover, the time of protein supplementation differs between studies (from a few days to a few months). Thus, future research should include the quantitative and qualitative assessments of protein intake in the habitual diet and the analysis of physical activity level, which influences the metabolism of protein products.

## 5. Conclusions

Osteoporosis is a tremendous challenge for the current healthcare system. All patients should be under good medical care. Treatment and prevention of osteoporosis are equally important. Research results do not favor any source of protein in the diet, as it is more effective in treating osteoporosis, but protein sources should be as varied as possible. Patients may benefit from increasing the quantity of protein consumed. However, increased protein intake might negatively influence bone quality in patients undergoing a bed regime. The analysis of protein consumption in osteoporosis development should include evidence-based nutritional studies conducted using a reliable research method that provides a detailed diet assessment based on a seven-day dietary recall [61].

## Figures and Tables

**Table 1:** Influence of dairy product consumption on BMD and risk of osteoporotic fracture.

Impact of Dairy Product Consumption on BMD and Osteoporotic Fracture					
Author	N—Number of Included Studies	Year of Study	n—Number of Patients	Results of Meta-Analysis	Characteristics of the Analyzed Population
Shi et al., 2020 [33]	N = 6	1995 to 2012	n = 618	Dairy products => ↑ BMD and plays an important role in the prevention of osteoporosis after menopause.	Healthy postmenopausal women
Fabiani et al., 2019 [32]	N = 20 (total) N =10 (fracture risk) N = 10 (low BMD risk)	2010 to 2017	n = 276,624 (total) n = 257,010 (fracture risk) n = 19,614 (low BMD risk)	“Milk/dairy” and “healthy” dietary patterns => ↓ risk of low BMD “healthy” pattern => ↓ risk of fracture. “Meat/western” dietary pattern => ↑ risk of low BMD and fracture BMD.	Youth >10 years old and adults
Malmir et al., 2020 [38]	N = 34 (total) N = 13 (osteoporosis) N = 21 (hip fracture)	1989 to 2018	n = 431,545 (total): n = 9641 (osteoporosis) n = 11,904 (hip fracture)	↑ intake of dairy products does not reduce the risk of osteoporosis and hip fracture.	Adults (>18 years old)

BMD—bone mineral density; ↑ increase; ↓ decrease.

**Table 2:** Lifestyle recommendations for patients with osteoporosis.

Lifestyle Changes in Patients with Osteoporosis		
Recommendations	Study Conformation/Scientific Proof	References
Plant and animal protein	Plant and animal proteins have a comparable effect on BMC and BMD changes and increase IGF-1 levels, which is potentiated by soy products. A diet containing only plant products is deficient in calcium and vitamin. Dairy products, except as a source of complete protein, deliver additional beneficial components of calcium, phosphorus, and aromatic amino acids. The quantity of protein should correspond with RDA in a specific population (high protein intake >1.2 g/kg body mass/day ↑ risk of osteoporosis in postmenopausal women and women under bed rest (>1.45 g/kg body mass/day)	[9] [27] [38] [15,16]
Soy products	The beneficial influence of isoflavones on the estrogen-deficient bone.	[39]
Nuts, soy, legumes, fish, poultry, and eggs	Recommended for patients with kidney diseases (red and processed meats have an unbeneficial influence on kidney function)	[12,13]
Poultry, low-fat dairy, fish, soy, and legumes	Advisable in CVD (red and processed meats, high-fat dietary products, and eggs should be avoided).	[40]
Adequate to age physical activity	In older people, prevents osteoporosis development. Safe and beneficial in patients with osteoporosis and osteopenia. ↓ the potential negative impact of protein intake on bone mineralization.	[17] [41] [15]

↑ increase; ↓ decrease.

## Data Availability

All data are included in the manuscript.
